# Prevalence of diabetes mellitus and hypertension amongst the HIV-positive population at a district hospital in eThekwini, South Africa

**DOI:** 10.4102/phcfm.v13i1.2766

**Published:** 2021-09-29

**Authors:** Althea Rajagopaul, Mergan Naidoo

**Affiliations:** 1 Discipline of Family Medicine, Faculty of Health Sciences, University of KwaZulu-Natal, Durban, South Africa

**Keywords:** HIV, diabetes mellitus, hypertension, prevalence duration of treatment age, BMI, gender

## Abstract

**Background:**

Life expectancies of HIV-positive patients have been increasing with the rapid implementation of antiretroviral therapy (ART). This has led to an increase in comorbidities such as diabetes mellitus (DM) and hypertension (HT) amongst the HIV population. The burden of the non-communicable diseases (NCDs) such as DM and HT need to be quantified in order to ensure that patients receive optimal integrated care as patients often access care at different clinics compromising holistic care.

**Aim:**

The aim of the study was to determine the prevalence of DM and HT amongst the HIV-positive population.

**Setting:**

The study was conducted at Wentworth Hospital, a district facility in South Durban, KwaZulu-Natal.

**Methods:**

This cross-sectional study was undertaken to determine the prevalence of two NCDs, namely DM and HT in HIV-positive patients attending the ART clinic at a district hospital in the eThekwini district. We compared the socio-demographic and clinical profiles of those with and without comorbidities. A sample of 301 HIV-positive patients were administered a structured questionnaire.

**Results:**

Of the 301 patients, 230 (76.41%) had HIV only (95% confidence interval [CI]: 71.25–80.89) and 71 (23.59%) had HIV and at least one comorbidity, namely DM and/or HT (95% CI: 19.11-28.75). Hypertension was the most prevalent comorbidity. This study revealed that there was no association between the duration of ART and comorbidities. Older age and body mass index (BMI) were associated with comorbidities, whilst gender and ethnicity were not associated.

**Conclusion:**

Non-communicable diseases such as DM and HT do pose a burden for HIV-positive patients attending the ARV clinic at this district facility. This study highlights the definite need to plan for the increased burden of NCDs as HIV-positive patients live longer and gain weight.

## Introduction

Human immunodeficiency virus (HIV) and non-communicable diseases (NCDs) are major public health concerns.^1^ Globally it was reported in 2020 that 37.9 million people are living with HIV. It is reported that HIV is of pandemic proportions in sub-Saharan Africa (SSA), which accounts for 68% of the global burden and is the leading cause of disability and death.^2,3^ With the increased roll out of antiretroviral therapy (ART), health systems are faced with the global challenge of managing comorbidities amongst patients living and ageing with HIV. It is reported that there are several factors that increase the risk of HIV-positive patients acquiring non-communicable diseases (NCDs), which pose a major public health concern.^4^ According to The Joint United Nations Programme on HIV/AIDS (UNAIDS), HIV continues to be a major global public health concern, having claimed more than 39 million lives.^4^ People are living longer with effective ART.^5^ The number of patients with HIV, accessing healthcare resources is also increasing because of improved screening, earlier diagnosis and better methods of treatment, greater accessibility and acceptance of therapy. Human immunodeficiency virus infection is incurable; however, with potent treatment options available patients lead long productive lives with an increased risk of acquiring NCDs over time.

KwaZulu-Natal (KZN) has the highest prevalence rate of HIV (18.9%) amongst adults aged 15 to 49 years of age. In SSA, the prevalence of diabetes mellitus (DM) and hypertension (HT) amongst the HIV population is also significantly high. Hypertension is a common condition that often coexists with DM with both conditions together causing an increased risk of cardiovascular morbidity and mortality.

People that are HIV-positive have three risk factors for contracting NCDs, namely from the HIV infection itself, from the adverse metabolic effects of ART and from the risk associated with increasing age. Gender, ethnicity and socio-economic status have also been associated with increased risk of contracting NCDs.^5^ The relationships between ART exposure and NCDs are still not well established as some studies have demonstrated a relationship between exposure whilst others have not.^6,7^ However, these studies have been conducted in high-income countries (HICs).^6,7^ In SSA, there is growing attention to NCDs and the challenge faced by the dual burden of HIV and NCDs in a resource constrained environment. With the introduction of integrated care and the ideal clinic strategies, data on the burden will help in their implementation. The increased prevalence of HT and DM in HIV-positive patients is reported to be linked to the age and ART duration.^8^ The aim of the study was to determine the prevalence of DM and HT and determine the factors associated with these NCDs amongst the HIV population attending the ART clinic at a district hospital in the eThekwini district, KZN, (Wentworth Hospital [WWH]), South Africa.

## Methods

### Study design

This epidemiological hospital based cross-sectional study generated quantitative data by means of a structured pretested questionnaire. A chart review was also conducted to confirm comorbidities amongst the study population.

### Setting

The study was conducted at Wentworth Hospital, a district facility in South Durban, KZN and the sample size of 301 was estimated using a confidence level of 95% with a precision of 5% based on a study population of 1379 patients that collected ART at the hospital during July 2016.

The content of the research instrument was validated by consulting with experts in the ART clinic and a biostatistician. A pilot study was conducted amongst 10 participants at another eThekwini hospital. After the content validity and pilot study, minor adjustments were made to the study tool. Data from the pilot study were captured on a Microsoft Excel spreadsheet and analysed. The results from this analysis confirmed that it would adequately address the aim and objectives of the study.

### Study population and sampling strategy

Study participants included HIV positive men and women who attended the Wentworth Hospital ART clinic who were 18 years and older and consented to participate. All consecutive patients met the inclusion criteria (all patients who were HIV-positive, above 18 years of age and those who agreed to participate in the study were asked to participate). Patients were approached as they waited in line to be consulted by the doctor.

### Data collection

Questionnaires were translated into isiZulu for easy comprehension and each participant was given an information sheet and signed the consent form after an explanation of the study was provided and queries addressed. Data were collected from May 2017 until November 2017. If a patient refused, then the next consecutive patient in line was approached to participate. Fieldwork was conducted from Monday to Friday during the clinics operating hours from 08:00 to 16:00. Trained fieldworkers collected the data whilst patients were in the waiting rooms of the ART clinic. The variables included patients’ socio-demographic characteristics and clinical information. Piloting of the questionnaire was performed on 10 participants and minor adjustments were then made to the study tool. All questionnaires were checked to ensure that most questions were answered. Those who had a substantial amount of missing data were rejected. Questionnaires were completed but it was found that some patients chose not to answer many questions resulting in substandard information so the questionnaire was rejected and a new study participant was recruited. This occurred for 13 participants. Eventually 301 patients satisfactorily completed the questionnaire.

### Data analysis

Coded data from the patient questionnaire was entered on a Microsoft Excel 2010 spreadsheet. The data were then imported into the statistical software package STATA version 14 for data analysis. Prevalence was reported as percentages with 95% confidence intervals (CIs). A statistical significance level of *p* < 0.05 was used to test the hypotheses of association between selected risk factors and presence of comorbidities. Univariate and multivariable binary logistic regression analyses were conducted to assess associations of interest and odds ratios. Hypertension was defined according to the seventh report of the internationally recognised Joint National Committee as systolic blood pressure (BP) ≥ 140 millimetres of mercury (mmHg) and/or diastolic BP ≥ 90 mmHg on two or more occasions or currently taking medication for HT. Diabetes mellitus was defined as a random blood sugar of > 11.1 mmol/L or fasting blood sugar equal or greater than 7 mmol/L or being on DM medication.^9^

### Ethical considerations

The study was approved by the Biomedical Research Ethics Committee at the University of KwaZulu-Natal (BE645/16) and from the KwaZulu-Natal Department of Health and Wentworth Hospital. All participants were assured that anonymity and strict confidentiality was maintained during the entire duration of the study. Participation was voluntary and participants could withdraw at any time during the study period. Informed consent and questionnaires for patients were provided in both English and isiZulu; however, all patients opted to use the English questionnaire. Patients signed the informed consent prior to participation in the study.

## Results

### Demographic factors

Altogether 301 patients participated in the study. Their demographic features are shown in Table 1. The overall mean age was 41.6 years (standard deviation [s.d.] = 11.04) and the sample was predominantly female (62.5%) and African (92.4%). Unemployment was also high in this sample population (64.4%).

**TABLE 1:** Demographic characteristics of participants.

Variable		Gender					
		Female		Male		Total	
		*n*	%	*n*	%	*n*	%
Ethnicity	African	171	91.0	107	94.7	278	92.4
	Indian	1	0.5	1	0.9	2	0.7
	Mixed-race	10	5.3	3	2.7	13	4.3
	White	6	3.2	2	1.8	8	2.7

Total	188	100.0	113	100.0	301	100.0
Employed	Employed	60	33.0	41	36.3	101	34.2
	Unemployed	120	65.9	70	61.9	190	64.4
	Unknown	2	1.1	2	1.8	4	1.4
	Total	182	100.0	113	100.0	295	100.0
Education level	Primary	12	6.4	7	6.2	19	6.3
	Secondary	121	64.4	74	65.5	195	64.8
	Tertiary	13	6.9	4	3.5	17	5.6
	Missing	42	22.3	28	24.8	70	23.3
**Total**	**-**	**188**	**100.0**	**113**	**100.0**	**301**	**100.0**

The mean age (and s.d.) amongst the female participants was 41.2 (11.2 s.d.) years and amongst male participants was 42.2 (10.9 s.d.) years and the entire group was 41.6 (11.0 s.d.) years.

### Prevalence of comorbidities

Table 2 shows the frequencies of the various comorbidities and the average age of each of the comorbidity subgroups.

**TABLE 2:** Prevalence of comorbidities (*N* = 301).

Variable	*n*	%	95% CI for prevalence	Mean age	s.d.	95% CI for mean age
HIV only	230	76.4	71.3–81.8	39.1	10.2	37.8–40.5
HIV and DM	6	2.0	0.9–4.4	43.2	8.5	34.2–52.1
HIV and HT	55	18.3	14.3–23.1	49.4	9.7	46.8–52.0
HIV, DM and HT	10	3.3	1.8–6.1	53.9	9.8	46.960.9
HIV and at least one comorbidity	71	23.6	18.1–28.9	49.5	9.8	47.251.8

HIV, human immunodeficiency virus; CI, confidence interval; s.d., standard deviations; DM, diabetes mellitus; HT, hypertension.

In this population, 71 (23.59%) of the participants had one or more comorbidities. There was a statistically significant difference in age between the subgroups (*p* < 0.001). Those participants with HIV alone were significantly younger than those with HIV and HT (*p* < 0.001) and those with both HT and DM (*p* < 0.001). In this study, 35.5% of those over 40 years old had comorbidities.

The association between selected risk factors and presence or absence of any comorbidity was examined using binary logistic regression. These are tabulated in Table 3.

**TABLE 3:** Factors associated with comorbidities.

Independent variable	Unadjusted odds ratios				Adjusted odds ratios			
	*p*	OR	95% CI for OR		*p*	AOR	95% CI for AOR†	
			Lower	Upper			Lower	Upper
**Age**	< 0.001	1.10	1.07	1.13	< 0.001	1.10	1.07	1.14
**BMI**	< 0.001	1.07	1.03	1.10	0.04	1.04	1.00	1.08
**Months on ART**	0.68	1.00	0.96	1.01	-	-	-	-
**Gender**								
Male	0.85	0.95	0.55	1.65	-	-	-	-
Female	-	Reference	-	-	-	-	-	-
**Ethnicity**								
African people	-	Reference	-	-	-	-	-	-
Indian people	0.39	3.41	0.21	55.34	-	-	-	-
Mixed-race people	0.20	2.13	0.67	6.75	-	-	-	-
White people	0.88	1.14	0.22	5.78	-	-	-	-

OR, odds ratio; AOR, adjusted odds ratio; CI, confidence interval; BMI, body mass index; ART, antiretroviral therapy.

† , Adjusted for age and body mass index.

There was no association between the duration of ART and comorbidities (*p* = 0.76). Gender or ethnicity was not associated with comorbidities. Age (*p* < 0.001) and body mass index (BMI) (< 0.001) were positively associated with comorbidities in a multiple logistic regression model. The older the patient or greater the BMI, the higher the likelihood of comorbidities. For every 1-year increase in age, the odds of comorbidities increased by almost 10% and for every one-unit increase in BMI, the odds of comorbidities increased by 4%.

### Duration of antiretroviral therapy

**FIGURE 1:**
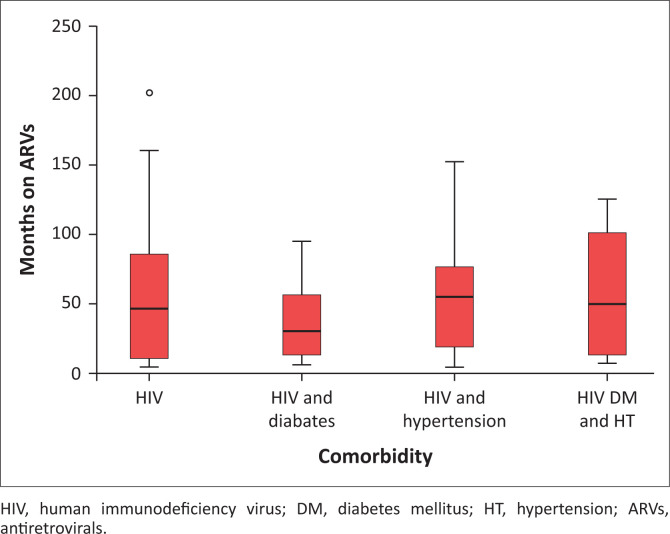
Box and Whisker plot for months on anti by comorbidities.

Duration of treatment was calculated in months between starting ARVs and the date of close of data extraction, that is, 11 August 2017. The results are depicted in Figure 1.

The time of treatment was skewed, with a mean of 52 months and median of 46 months. The range was from 1.9 to 201 months. A non-parametric Kruskal–Wallis test was used to test the null hypothesis that there was no difference in months on ARVs between the groups.

## Discussion

The key findings were that the prevalence of comorbidities in this population was 23.59% (95% CI: 19.1–28.9). Older age and BMI were positively associated with comorbidities (*p* < 0.001 and *p* = 0.040, respectively) and there was no association between the duration of ART and comorbidities (HT and DM).

Hypertension prevalence amongst the HIV-positive population varies between 6.0% and 39.0% and this figure is consistent with our study.^10^ Cameroon and Tanzania reported a HT prevalence of 38.0%^11^ and 28.7%,^12^ respectively. A study conducted in Johannesburg reported a HT prevalence rate of 19.1% amongst the HIV-positive population, which is similar to our study.^13^ A study conducted in South America in various Latin American countries such as Brazil, Peru and Chile reported a HT prevalence of 31.5%.^14^ A study carried out in Uganda reported a 28.0% prevalence of HT.^15^

Diabetes mellitus prevalence in the HIV population is emerging as the major non-infectious comorbidity.^5^ The prevalence of type 2 DM (T2DM) has been reported as five- to nine-fold greater in HIV-positive individuals than in HIV-negative individuals.^16^ The prevalence of DM was 8% (95% CI: 5.5–10.5)^5^ in the study conducted in Ethiopia, which is similar to our findings. The estimated prevalence of DM amongst HIV patients range from 2% to 14%.^17^ Some investigators reported HIV as a risk factor for DM^18,19^ but others showed no association with DM.^20,21^

Various studies reported a prevalence of DM and HT as being higher in the population with HIV compared with the general population.^1,2^ The prevalence of comorbidities in our study was similar to the reported prevalence of 29% in the Brent population of London.^22^ The prevalence of DM and HT in Brent, Senegal; Northwestern Tanzania and Southern Uganda was reported as 8% (95% CI: 4.1–11.9), 15.9 % (95% CI: 10.5–21.3),^7^ 14.5% (95% CI: 10.3–19.5) and 28.1% (95% CI: 22.5–34.2),^8^ respectively.

Diabetes mellitus prevalence was associated with older age, increased BMI and longer duration of treatment in the study in Ethiopia.^23^ However, unlike HIV-hypertension comorbidity ART regimens containing Tenofovir disoproxil fumarate (TDF) increased the risk of HIV-diabetes comorbidity.^23^ Certain metabolic factors related to HIV, and to HIV therapy, may increase the incidence of DM.^24^ Body mass index was significantly associated with HT (95% CI: 1.26–5.84) in the study in Tanzania^1^ whilst a study in Cameroon reported a BMI of 24.8 in patients with HT and HIV.^25^ Another study in Tanzania reported a similar HT prevalence of 26.2% with high BMI (adjusted odds ration [AOR]: 2.37; 95% CI: 1.26–5.84).^1^ Increased BMI increases the risk of HIV patients developing DM.^18^ Increasing the duration of patients on ART increases the risk of gaining weight and the risk factor for NCDs.

The Tanzanian study revealed an increase in the prevalence of obesity from 33.0% to 58.0% after commencement of ART.^1^ There was no correlation between BMI and duration on ART in our study and this may be because of the shorter mean duration of ART. In the veterans ageing cohort study of 3227 HIV-positive individuals, the baseline prevalence of T2DM was 14.9% in the HIV-positive group as compared with 21.4% in the HIV-negative group with the difference being attributed to the differences in BMI.^26^

Older age is an independent risk factor for NCDs in the HIV population.^1,15,27^ Tanzanian patients had a median age of 40^1^ and Cameroonian patients had a mean age of 40.2 years with older age (40 and above) associated with an increased prevalence of DM and HT in the HIV population.^8^ The Dimalas study^11^ reported that 23.1% of patients above the age of 40 years had comorbidities.^11^ In a cohort study conducted in Sweden, DM was observed amongst the HIV-positive patients who were 60 years of age and older.^28^ This is similar to the HIV-negative population who show similar prevalence rates of DM in patients older than 40.^24^ The prevalence of HT amongst the HIV population increased with age however it was the same as the trend seen on negative patients. Patients older than 50 years were 2.35 times more at risk of HT compared with patients who were 40 years or younger. It was reported as a global estimate that approximately 5% – 15% of HIV-negative adults aged < 40 years had HT whilst HIV patients reported prevalence 18.6%.^29^

In our study, duration of ART was not associated with comorbidities but this was not consistent with other studies.^8,11,30^ Studies have reported concerns relating to complications with the use of ART.^31^ In Nigeria, an increase in the prevalence of HT from 26.0% to 31.7% was reported after two years of ART.^32^ A Spanish study found and an increase in blood pressure after 48 weeks of exposure to ARTs, however, the study sample size was very small and no significant association with HIV populations was made.^33^ Xu et al. highlighted in their study that in the same group of HIV patients who reported a HT prevalence of 18.6%, HT prevalence increased further to 23.5% for patients on ART.^29^ However, one study showed that patients presented with HT prior to commencement of ART.^34^ A study in South Africa found that only four patients developed DM following the duration of treatment.^13^ This could be attributed to the fact that patients in South Africa are mostly on the ART drug combination, which excludes protease inhibitors (PIs). Antiretroviral treatment is known to cause metabolic dysfunction by causing insulin resistance, DM, dyslipidemia and lipodystrophy and these are mostly associated with PI-based regimens.^35,36^ Regimens that include efavirenz, stavudine and zidovudine have all been shown to be associated with increased incident of diabetes.^37^ Another study conducted on 755 HIV-positive individuals attending tertiary care hospital for routine clinical and laboratory follow-up between July 2009 and September 2009 in Italy, reported a DM prevalence as 4.5%.^18^ This study concluded that a longer duration of ART was significantly associated with the onset of DM. It was reported that patients on HIV treatment were two times at risk of developing DM.^11^ Dimala found no direct association between HT, DM and ART exposure but did suggest future studies to be conducted in SSA to find any significant association.^38^

Data are available regarding metabolic syndrome and gender on patients on HIV treatment.^3^ It was reported that females on ART had a higher prevalence rate of HT^7^ whilst another study found males on ART to have a higher prevalence.^38^ It was reported that DM and HT are more prevalent amongst certain ethnic groups being more common amongst the African population.^39^ In the United States, HT was more prevalent (46%) amongst the African American population, which was more than other ethnic groups.^40^ There is a paucity of data reporting the association between ethnicity and the prevalence of DM and HT amongst patients living with HIV. Ethnicity was also not a significant risk factor for comorbidities in this study as the various ethnic groups were not equally represented. An epidemiological study conducted showed that people of South Asian, African, and African Caribbean origins have a higher prevalence of DM when compared with the general population.^37^ In 2000, after adjusting for age in a prevalence study, it was reported that in the adult population of Durban, HT was the highest in black people (25%), intermediate in white people (17.2%) and the lowest in Indians (14.2%). This was because of the catchment area of the facility under study serviced predominately the African and mixed ethnicity population.

### Strengths and limitations

One of the strengths of this study is that this is one of few studies to describe the prevalence of DM and HT amongst the HIV population in an urban district hospital setting. One of the limitations of the study is that the results of this study cannot be generalised to the population at large because of the small sample size, cross-sectional design and being conducted in one study site. Another limitation in our study was that as the most stable patients on treatment were down referred to primary health care (PHC) clinics hence the shorter duration of treatment in the study population. Data on ART regimens were not collected and 13 questionnaires were rejected and new participants enrolled because of substandard data collection. This facility serviced predominately the black population and mixed race population and inferences cannot be made for the population at large.

### Recommendations

Health promotion messages need to be incorporated into routine care as HIV-positive patients live and age with HIV. Sometimes health promotion in the ART clinics is neglected and priority is given to adherence to HIV treatment. It is important that NCDs and HIV is managed at one point of care as fragmented care comprises the quality and continuity of care as patients are observed to prioritise NCDS over HIV or vice versa. It is therefore important to integrate HIV and NCD clinics where patients are managed for all their conditions by trained clinicians upskilled in ART and NCDs treatment.

## Conclusion

Non-communicable diseases such as DM and HT add to the burden of the HIV patients attending this district facility. Older age and increased BMI were predictors of comorbidities amongst the HIV population in our study. It was highlighted that almost a quarter of the patients who presented at this ART clinic had a comorbidity. Based on the findings of this study, it is imperative that health awareness and ongoing health education is conducted to highlight the NCDs risk factors and self-management of risk factors. It is important for clinicians involved in HIV care to note the importance that age and BMI play in increasing the odds of developing NCDs. Screening for NCDs need to be an integral part of the follow up routine care of HIV-positive patients and all patients that enter healthcare facilities.
